# Differences in genetic population structures of *Plasmodium falciparum *isolates from patients along Thai-Myanmar border with severe or uncomplicated malaria

**DOI:** 10.1186/1475-2875-7-212

**Published:** 2008-10-21

**Authors:** Pannapa Susomboon, Moritoshi Iwagami, Noppadon Tangpukdee, Srivicha Krusood, Sornchai Looareesuwan, Shigeyuki Kano

**Affiliations:** 1 Department of Appropriate Technology Development and Transfer Research Institute, International Medical Center of Japan, 1-21-1Toyama, Shinjuku, Tokyo 162-8665, Japan; 2 Faculty of Tropical Medicine, Mahidol University, 420/6 Ratchawithi Rd, Bangkok 10400, Thailand

## Abstract

**Background:**

There have been many reports on the population genetic structures of *Plasmodium falciparum *from different endemic regions, but few studies have examined the characteristics of isolates from patients with different clinical outcomes. The population genetic structures of *P. falciparum *isolates from patients with either severe or uncomplicated malaria were examined.

**Methods:**

Twelve microsatellite DNA loci from *P. falciparum *were used to assess the population genetic structures of 50 isolates (i.e., 25 isolates from patients with severe malaria and 25 from patients with uncomplicated malaria) collected in the Thai-Myanmar border area between 2002 and 2005.

**Results:**

Genetic diversity and effective population sizes were greater in the uncomplicated malaria group than in the severe malaria group. Evidence of genetic bottlenecks was not observed in either group. Strong linkage disequilibrium was observed in the uncomplicated malaria group. The groups demonstrated significant genetic differentiation (*P *< 0.05), and allele frequencies for 3 of the 12 microsatellite loci differed significantly between the two groups.

**Conclusion:**

These findings suggest that the genetic structure of *P. falciparum *populations in patients with severe malaria differs from that in patients with uncomplicated malaria. The microsatellite loci used in this study were presumably unrelated to antigenic features of the parasites, but, these findings suggest that some loci may influence the clinical outcome of malaria.

## Background

*Plasmodium falciparum *is the most pathogenic of the protozoan parasites that cause human malaria. According to the World Health Organization, more than 1 million people die from malaria each year [1]. The clinical manifestations of malaria are quite pleomorphic, ranging from mild or asymptomatic parasitemia to potentially fatal conditions such as coma and multi-organ failure. Although the molecular basis of severe malaria has been well studied in recent years [2], determinants of the clinical outcomes of malaria remain unknown. Several factors, including host and parasite genetic characteristics, are thought to contribute to the clinical outcome of malaria.

Several studies have reported evidence of a relationship between parasite genotype and clinical outcome [2-8]. Ariey *et al *found that certain alleles in the polymorphic microsatellite loci of clinical isolates from French Guyana were associated with disease severity [6]; however, Ferreira *et al *found no evidence to suggest that the parasite genotypes of the isolates from malaria patients in Vietnam were associated with disease severity [8]. These results demonstrate the difficulties associated with evaluating the role of parasite genetic factors in malaria pathogenesis.

The present study examines the relationship between parasite genetic factors and clinical outcomes. To this end, the genetic population structures of parasites isolated from patients with either uncomplicated or severe malaria were compared using highly polymorphic microsatellite DNA loci from *P. falciparum*.

## Methods

### Sample collection

A total of 50 *P. falciparum *isolates were collected from patients who contracted malaria along the Thai-Myanmar border and who had been admitted to the Bangkok Hospital for Tropical Diseases, Faculty of Tropical Medicine, Mahidol University between 2002 and 2005. Inclusion criteria of the study are: age ≥ 15 years old, both male and female, positive for *P. falciparum *malaria infection with microscopic confirmation showing acute manifestation, informed consent to take part in the study obtained either from the patient or the legal guardian, and signs of severe malaria according to the World Health Organization criteria [9] (for patients in severe malaria group). Exclusion criteria are: pregnant or lactating women, pre-treatment with any anti-malarial drugs within the past two weeks, evidence of mixed infection on admission, and the evidence of any contra-indication of blood sampling collection. Consequently, the 50 isolates were divided into two clinical groups (25 isolates from patients with severe malaria and 25 from patients with uncomplicated malaria) (Additional File 1, 2). Hyperparasitemia (> 250,000 parasite/μL), peripheral schizontemia, blood urea nitrogen (> 60 mg/dL) and elevation of serum enzymes (AST, ALT) were noted for patients in the severe group. Clinical manifestations such as nausea/vomiting and jaundice were also taken into account of the clinical severity of the patients.

The geographic and temporal distributions of the presumed area of infection were similar among both groups. Blood samples and clinical data were collected from patients after obtaining informed consent. Blood samples were frozen at -80°C until use.

This study was approved by the Ethics Committee of the Faculty of Tropical Medicine at Mahidol University in Thailand, and complied with the ethical guidelines for epidemiological studies set forth by the Japanese Ministry of Education, Culture, Sports, Science, and Technology and the Japanese Ministry of Health, Labour, and Welfare.

### Preparation of parasite genomic DNA and genotyping analyses

Genomic DNA was extracted from the parasites in 2 mL of frozen blood sample using previously described methods [10].

### Genotyping

Twelve microsatellite DNA loci were amplified by semi-nested PCR. The loci were as follows: TA1 (Chromosome 6), TA40 (Chromosome 10), Poly α (Chromosome 4), TA60 (Chromosome 13), ARAII (Chromosome 11), Pfg377 (Chromosome 12), PfPK2 (Chromosome 12), TA109 (Chromosome 6), TA87 (Chromosome 6), TA81 (Chromosome 5), TA42 (Chromosome 5), and 2490 (Chromosome 10). The PCR primer sets and amplification conditions were consistent with the protocol of Anderson *et al*, using a modified TA40 primer set [11,12]. Sizes of fluorescence-labeled PCR products were measured on an Applied Biosystems Prism Genetic Analyzer 310 using Gene Scan version 3.1.2 with a 500 ROX size standard (ABI, CA, USA).

Different-sized PCR products amplified using the same primer set were considered to be individual alleles within a locus, as size variation among isolates is consistent with the repeat number in a microsatellite locus.

### Data analysis

Expected heterozygosity (*H*) was calculated for each locus based on the allele frequencies of the 12 examined microsatellite loci. *H *values were calculated using *H *= [n/(n - 1)] [1 - ∑*pi*^2^], where n corresponds to the number of isolates examined and *pi *is the frequency of the *i*th allele.

Effective population size (*Ne*) was estimated based on *H *and the microsatellite mutation rate (*μ *= 1.59 × 10^-4^; 95% confidence interval: 6.98 × 10^-5^, 3.7 × 10^-4^) for *P. falciparum *[13-15]. The infinite-alleles model (IAM) and the stepwise mutation model (SMM) were used to estimate *Ne*.

Each population was examined for evidence of a recent genetic bottleneck (i.e., a severe decrease in population size). In non-bottlenecked populations approaching mutation-drift equilibrium, the *H *value for each locus was calculated based on the number of alleles, and the sample size was equal to the observed Hardy-Weinberg equilibrium heterozygosity (*He*) [16,17]. After a bottleneck event, the number of alleles and the expected heterozygosity of a population are predicted to decrease; however, rare alleles are purged more quickly, reducing the number of alleles without altering the extent of heterozygosity [16,17]. Furthermore, the mode shift in allele frequency distribution for the presence of rare alleles was examined. The BOTTLENECK program (version 1.2.02) [18] was used to search for evidence of heterozygosity excess and mode-shift. The Sign Test and the Wilcoxon Signed-Rank Test were used to evaluate statistical significance.

Multilocus linkage disequilibrium was assessed using the standardized index of association (*I*_A_^S^) [19,20]. This analysis was performed using the LIAN 3.5 Web interface [21]. *I*_A_^S ^was calculated using the formula *I*_A_^S ^= (*V*_D_/*V*_e _- 1)/(*l *- 1) with permutation testing of the null hypothesis of complete linkage equilibrium (*I*_A_^S ^= 0), where *V*_D _is the observed mismatch variance, *V*_e _is the expected mismatch variance, and *l *is the number of examined loci. Significances of the observed *I*_A_^S ^values were calculated by Monte-Carlo simulation, using 10,000 random permutations of the data. This statistic is a variation of the method proposed by Maynard-Smith *et al*. To enable comparison of different data sets, the results were standardized by the number of loci [19,22].

The extent of population subdivision was estimated using Weir and Cockerham's theta estimator for determining F statistics (*F*_*ST*_) [23]. *F*_*ST *_were calculated using the FSTAT program (version 2.9.3.2: available at ) [24] and tested for significant difference from 0, based on 1,000 random permutations of the data set.

Fisher's exact test was used to calculate differences in allele frequencies for each locus in the uncomplicated and severe malaria groups (SAS for Windows, version 9.1.3; SAS Institute Inc., Cary, NC, USA). A *p*-value of less than 0.05 was considered statistically significant.

## Results

Allele frequencies for each locus are shown in Additional File 3. Of the 50 isolates examined, 45 (i.e., 90%) represented single-genotype infections involving all 12 loci. The remaining five isolates (i.e., 10%) represented multiple-genotype infections involving one to four loci. Three of the five isolates were obtained from the severe malaria group, while the remaining two isolates were obtained from the uncomplicated malaria group. Data from multiple loci were excluded from the analyses. We excluded data from loci that originated from multi-genotype infection.

### Genetic diversity

The genetic diversity of each population was assessed by determining the number of alleles per locus in each population and by calculating the expected *H *values (Table 1). The mean numbers of alleles ± SE in the severe and uncomplicated malaria populations were 5.17 ± 0.66 and 6.58 ± 0.66, respectively. The *H *values ± SE for the severe and uncomplicated malaria populations were 0.60 ± 0.07 and 0.71 ± 0.05, respectively. These results indicated that the genetic diversity in the uncomplicated population was slightly greater than that in the severe population, although the differences were not statistically significant.

**Table 1:** Sample size, mean number of alleles, and expected heterozygosity in the two study populations.

Population	No. of isolates	No. of alleles	Expected heterozygosity (*H*)
	mean ± SE	mean ± SE	mean ± SE

Severe malaria	24.50 ± 0.15	5.17 ± 0.66	0.60 ± 0.07

Uncomplicated malaria	24.67 ± 0.19	6.58 ± 0.66	0.72 ± 0.05

### Effective population size

*Ne *values were calculated from the mean expected heterozygosity and mutation rates of *P. falciparum *microsatellite loci using the infinite-allele model (IAM) and the stepwise mutation model (SMM) (Table 2) [14,15]. The sizes of the severe and uncomplicated malaria populations were 2,356 and 3,944, respectively, based on IAM, and 4,120 and 8,890, respectively, based on SMM. These findings indicated that the severe malaria population was genetically less divergent than the uncomplicated malaria population.

**Table 2:** Effective size (*Ne*) values for the two study populations.

Population	IAM	SMM
Severe malaria	2,356 (1,012, 5,366)	4,120 (1,770, 9,385)
Uncomplicated malaria	3,944 (1,695, 8984)	8,890 (3,820, 20,251)

### Genetic bottleneck

Evidence of genetic bottleneck was assessed based on heterozygosity excess and patterns of allele frequency distribution (i.e., mode-shift) [16,17]. Table 3 shows the number of loci corresponding to *H *excess and deficiency. Statistically high levels of *H *excess compared with *H *deficiency were not observed in either study population when IAM and SMM were applied to the analyses, indicating the absence of genetic bottleneck events. The mode-shift indicator test revealed a normal L-shaped allele frequency distribution in the two study populations (Table 3), further demonstrating the absence of genetic bottleneck events.

**Table 3:** Observed versus expected heteroxygosity in the study populations.

		IAM			SMM			
				
Population	No. of loci	*H *excess	*H *deficiency	*P*	*H *excess	*H *deficiency	*P*	Mode- shift
Severe malaria	12	8	4	NS	5	7	NS	normal
Uncomplicated malaria	12	6	6	NS	2	10	*P *< 0.05	normal

Proportion of loci with excessive or deficient observed heterozygosities, relative to expected heterozygosities. Data were subject to mutation drift equilibrium via IAM and SMM, as well as mode-shift analyses. H: heterozygosity. NS: not significant.

### Multilocus linkage disequilibrium

*I*_*A*_^*S *^values were calculated for 12 loci from 22 isolates in the severe malaria group and 23 isolates in the uncomplicated malaria group. *I*_*A*_^*S *^values were equal to 0.010 and 0.127 for the severe and uncomplicated groups, respectively (Table 4). Significant linkage disequilibrium was observed in the uncomplicated malaria population (*p *< 0.0001).

**Table 4:** Multilocus linkage disequilibrium in the two study populations.

Population	No. of loci	No. of isolates	*I*_A_^S^
Severe malaria	12	22	0.010
Uncomplicated malaria	12	23	0.127**

**P < 0.0001

### Genetic differentiation and allele frequency-distribution patterns

**Figure 1:**
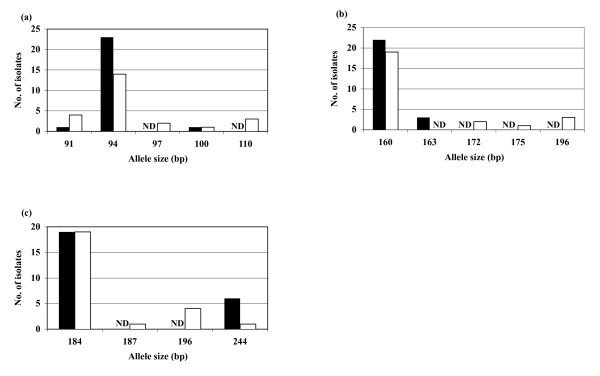
**Allele frequency distribution of the three microsatellite loci Pfg377 (a), TA109 (b), and TA42 (c).** Black bars represent the severe malaria group; white bars represent the uncomplicated malaria group. ND: not detected.

Genetic differentiation between the two populations was indicated by an *F*_*ST *_value of 0.014. This value was found to be significantly different from 0 (*p *< 0.05). The distribution of alleles for each locus in the severe malaria group was compared with that in the uncomplicated group. Distribution patterns for 3 (i.e., Pfg377, TA109, and TA42) of the 12 loci were significantly different (*p *< 0.05) between the two populations (Figure 1). Specifically, distribution patterns in the uncomplicated malaria population were more variable than those in the severe malaria population.

## Discussion

The genetic structures of two parasite populations collected from patients with either severe or uncomplicated malaria who contracted malaria, from the Thai-Myanmar border region were assessed. This area is known to be endemic for multidrug-resistant malaria [25-27]; transmission is unstable and hypoendemic [28]. However, morbidity and mortality in the area have gradually decreased in recent years, with a 2006 parasite incidence of 0.49 per 1,000 people [29].

In the present study, only 10% of the isolates were obtained from multi-genotype infections involving one to four loci. This is significantly lower than a previous estimate (i.e., 40%) reported for the same region in 1997 and 1998 [22]. The frequency of multiple-genotype infection by malaria parasites is known to increase as malaria transmission becomes more prevalent. For example, more than 50% of isolates are obtained from multiple-genotype infection in high-transmission areas (e.g., Uganda, Congo, and Zimbabwe), while fewer than 10% are obtained from multiple-genotype infection in low-transmission areas (e.g., Colombia and Brazil) [22]. Therefore, the present study indicates that malaria transmission has decreased in the Thai-Myanmar border region in recent years.

The genetic structure of the *P. falciparum *population in the severe and uncomplicated malaria groups differed in regards to several genetic indexes. Specifically, *H *values in the severe population were lower than those in the uncomplicated population. *H *values determined for both the severe and uncomplicated groups were higher than previously reported values for the same area [22]. *Ne *values for the severe population were also lower than those in the uncomplicated population, and our values were generally higher than previously reported values for this area [22]. Discrepancies between our study and previous reports may have resulted from differences in the length of sampling periods. In the present study, samples were collected between 2002 and 2005, while other investigators collected samples between December 1997 and January 1998. Thus, the longer sampling period used in our study may have resulted in higher *H *and *N*e values in the *P. falciparum *populations.

Significant genetic differentiation was observed between the severe and uncomplicated malaria groups (*p *< 0.05), even though the groups were collected from the same endemic area. This may reflect differences in transmission intensity between the two groups.

Allele frequency distributions for 3 (i.e., Pfg377, TA109, and TA42) of 12 loci differed significantly between the uncomplicated and severe malaria groups (*P *< 0.05). Pfg377 is located within the telomeric region of chromosome 12, while TA109 is located within a hypothetical protein on the centromeric region of chromosome 6, and TA42 is located within a hypothetical protein on the telomeric region of chromosome 5.

Differences in allele frequencies between the severe and uncomplicated malaria groups are mainly attributed to variations in minor alleles among the respective groups. The sizes and frequencies of the major alleles were similar in the two groups, while the numbers of minor alleles in the uncomplicated group were higher than those in the severe group.

In a previous study of the Western Amazon, Martha *et al *found that allele frequency distribution within the TA42 locus differs between symptomatic and non-symptomatic malaria patients [7]. In light of these results, the authors suggested that specific alleles within the TA42 locus are dominant in non-symptomatic malaria patients; however, evidence of such phenomena was not observed in either the severe or uncomplicated populations.

Other possibilities that are not linked with the parasite genetic background and that could influence the population structure, such as age of the patient, or number of days between onset of the symptom and admission to the hospital, were excluded by the statistical analysis of the two groups (Additional File 1, 2).

Many population geneticists have accepted a model of *P. falciparum *infection that depicts low-transmission regions as having high levels of monoclonal infection, inbreeding, and rare recombination; while high-transmission regions are depicted as having frequent mosquito inoculation, multiple-genotype infections, frequent outbreeding, and extensive recombination [22]. According to this model, genetic diversity increases in high-transmission regions and decreases in low-transmission regions. The results described in the present study appear to link genetic diversity with transmission levels in both the severe and uncomplicated populations. Luxemburger *et al *found that only 5% of malaria cases in the Thai-Myanmar border region in 1992 were severe: the remainder was uncomplicated [30].

The genetic indexes examined in this study were generally consistent with transmission levels; however, the level of linkage disequilibrium in the severe malaria group was lower than that in the uncomplicated malaria group. For reasons that remain unclear, significant linkage disequilibrium was evident in the uncomplicated malaria group but not in the severe group. Further studies are required to explore this finding in greater detail.

## Conclusion

Twelve highly polymorphic microsatellite loci were examined and the genetic structure of *P. falciparum *populations in patients with severe malaria was demonstrated to differ from that in patients with uncomplicated malaria. The microsatellite loci used in this study were presumably unrelated to the antigenic features of the parasites; however, it was suggested that the loci might somehow influence the clinical outcome of malaria.

## Competing interests

The authors declare that they have no competing interests.

## Authors' contributions

PS and MI carried out the molecular genetic studies, performed the population genetic analysis and drafted the manuscript. NT, SKr and SL performed the clinical management and collected the patients' blood samples as well as helping with the writing of the manuscript. SKa participated in the design of the study, acquisition of funding, coordination and writing of the manuscript.

## Supplementary Material

Additional file 1 Clinical data of 25 severe malaria patients at time of admission. RBC, red blood cell; Hb, hemoglobin; Ht, hematocrit; WBC, white blood cell; Plt, platelet; BUN, blood urea nitrogen; Cr, creatinine; Alb, albumin; AST, aspartate aminotransferase; ALT, alanine aminotransferase; PCT, parasite clearance time; FCT, fever clearance time. Click here for file

Additional file 2 Clinical data of 25 uncomplicated malaria patients at time of admission. RBC, red blood cell; Hb, hemoglobin; Ht, hematocrit; WBC, white blood cell; Plt, platelet; BUN, blood urea nitrogen; Cr, creatinine; Alb, albumin; AST, aspartate aminotransferase; ALT, alanine aminotransferase; PCT, parasite clearance time; FCT, fever clearance time; NA, not available. Click here for file

Additional file 3 Allele frequencies and number of isolates (n) of loci within the two study populations. Click here for file
